# The Ameliorative Effect of Sophoricoside on Mast Cell-Mediated Allergic Inflammation *in Vivo* and *in Vitro*

**DOI:** 10.3390/molecules18056113

**Published:** 2013-05-22

**Authors:** Su-Jin Kim, Gil-Yong Lee, Ji-Wook Jung, Sa-Rang Oh, Eun-Mi Ahn, Sung-Hoon Kim, Seung-Heon Hong, Jae-Young Um

**Affiliations:** 1 Department of Cosmeceutical Science, Daegu Hanny University, Yugok-dong, Kyungsan 712-715, Korea; E-Mail: ksj1009@dhu.ac.kr; 2 Department of Herbal Medicinal Pharmacology, Daegu Haany University, Yugok-dong, Kyungsan 712-715, Korea; E-Mails: leebssong@nate.com (G.-Y.L.); jwjung@dhu.ac.kr (J.-W.J.); 3 Department of Pharmacy, Keimyung University, Sindang-dong, Dalseo-gu, Dae-gu 704-701, Korea; E-Mail: blazma1021@nate.com; 4 Department of Herbal Foodceutical Science, Daegu Haany University, Yugok-dong, Kyungsan 712-715, Korea; E-Mail: ahnem@dhu.ac.kr; 5 College of Korean Medicine, Institute of Korean Medicine, Kyung Hee University, 1 Hoegi-Dong, Dongdaemun-gu, Seoul 130-701, Korea; E-Mail: sungkim7@khu.ac.kr; 6 Department of Oriental Pharmacy, College of Pharmacy, Wonkwang University, Jeonbuk 570-749, Korea; E-Mail: jooklim@wonkwang.ac.kr

**Keywords:** sophoricoside, inflammatory cytokine, mast cells, allergic inflammation

## Abstract

Sophoricoside exhibits numerous pharmacological effects, including anti- inflammatory and anti-cancer actions, yet the exact mechanism that accounts for the anti-allergic effects of sophoricoside is not completely understood. The aim of the present study was to elucidate whether and how sophoricoside modulates the mast cell-mediated allergic inflammation *in vitro* and *in vivo*. We investigated the pharmacological effects of sophoricoside on both compound 48/80 or histamine-induced scratching behaviors and 2,4-dinitrochlorobenzene (DNCB)-induced atopic dermatitis in mice. Additionally, to find a possible explanation for the anti-inflammatory effects of sophoricoside, we evaluated the effects of sophoricoside on the production of histamine and inflammatory cytokines and activation of nuclear factor-κB (NF-κB) and caspase-1 in phorbol 12-myristate 13-acetate plus calcium ionophore A23187 (PMACI)-stimulated human mast cells (HMC-1). The finding of this study demonstrated that sophoricoside reduced compound 48/80 or histamine-induced scratching behaviors and DNCB-induced atopic dermatitis in mice. Additionally, sophoricoside inhibited the production of inflammatory cytokines as well as the activation of NF-κB and caspase-1 in stimulated HMC-1. Collectively, the findings of this study provide us with novel insights into the pharmacological actions of sophoricoside as a potential molecule for use in the treatment of allergic inflammation diseases.

## 1. Introduction

Atopic dermatitis (AD) is a chronic inflammatory skin disease characterized by eczematous inflammation of the skin [1]. The incidence of this disease has increased steadily over recent years. AD is known to be the result of an immune system dysregulation, ultimately resulting in allergic inflammation [2].

Mast cells are tissue-based inflammatory cells of bone marrow origin, which respond to the danger signals of innate and acquired immunity with both immediate and delayed releases of inflammatory mediators. It has been previously reported that mast cells can be found in larger numbers in AD lesional skin. In response to various stimuli, mast cells generate a variety of cytokines and these cytokines may be of major importance in the development of a variety of inflammatory skin disorders [3]. Therefore, the inhibition of cytokine secretion can aid in the development of a useful therapeutic strategy for allergic inflammatory diseases such as AD.

Nuclear factor-kappa B (NF-κB) plays a critical role in the expression of many of the genes involved in immune and inflammatory responses [4]. In the nucleus, NF-κB activates gene transcription; thus, NF-κB performs a pivotal function in the regulation of immune and inflammatory responses, occurring via the control of the transcription of inflammatory cytokine genes [5]. An increase in NF-κB activity associated with the secretion of high levels of interleukin (IL)-6 and tumor necrosis factor (TNF)-α has also been noted in the context of allergic inflammatory responses [6]. The results of those studies demonstrated that NF-κB activation and the subsequent activation of pro-inflammatory cytokine gene expression are critically important in the initiation and perpetuation of allergic inflammation. Caspase-1, originally designated IL-1 converting enzyme, is a member of a family of caspases with large prodomains [7,8]. Activation of caspase-1 induces inflammatory response via the production of pro-inflammatory cytokines and the recruitment of neutrophils [9]. These results have implicated caspase-1 activation as an attractive target for the treatment of inflammatory diseases.

**Figure 1:**
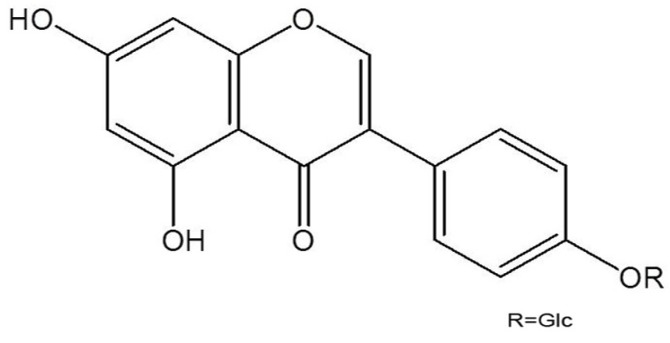
Chemical structure of sophoricoside.

Sophoricoside is an isoflavone glycoside (Figure 1) isolated from *Sophora japonica*, a plant of the Leguminosae family. Numerous pharmacological effects of sophoricoside have been reported, including anti-inflammatory, anti-cancer and immunosuppressive effects [10,11]. For example, sophoricoside has cytotoxic activity in human breast cancer cells and inhibits the allergic inflammation through regulation the IL-5 production [12,13]. Sophoricoside is also efficient in inhibition of ovariectomy-induced bone loss [14]. However, the precise molecular mechanisms of sophoricoside on mast cell-mediated allergic inflammation have yet to be clearly elucidated. 

In this study, we attempted to ascertain the mechanisms underlying the pharmacological effects of sophoricoside on both compound 48/80 or histamine-induced scratching behaviors and 2,4-dinitrochlrobenzene (DNCB)-induced atopic dermatitis in mice. Additionally, to find a possible explanation for the anti-allergic mechanisms of sophoricoside, we evaluated the effects of sophoricoside on the production of histamine and inflammatory cytokines and activation of NF-κB and caspase-1 in phorbol 12-myristate 13-acetate plus calcium ionophore A23187 (PMACI)-stimulated human mast cells (HMC-1).

## 2. Results and Discussion

### 2.1. Effect of Sophoricoside on Scratching Behaviors in Mice

**Figure 2:**
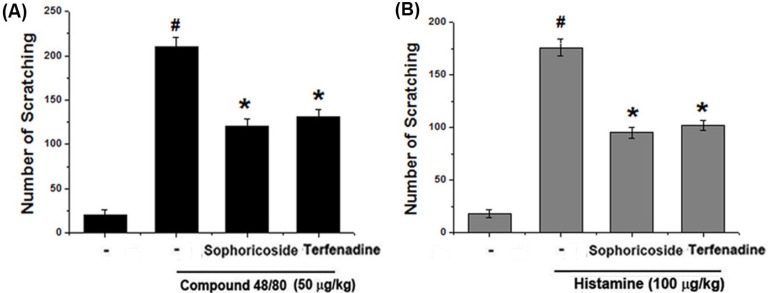
Effect of sophoricoside on scratching behavior in ICR mice. (**A**,**B**) Sophoricoside (2 mg/kg) was orally administered 1 h before compound 48/80 (50 µg/kg) or histamine (100 µg/kg) intradermally injection. Scratching behaviors was counted as one incident of scratching for 30 min. Terfenadine (10 mg/kg) was used as a positive control. Each datum represents the means ± S.E.M. of three independent experiments (^# ^*p* < 0.05 *vs.* control group, * *p* < 0.05 *vs.* compound 48/80 or histamine-treated group).

The anti-pruritic effects of sophoricoside were investigated on the compound 48/80-induced scratching behavior animal model. When the sophoricoside was orally administered 1 h before compound 48/80 injections, the scratching behaviors was reduced. The inhibition rate of sophoricoside (2 mg/kg) was approximately 41.21% (Figure 2A). In addition, we investigated the contribution of sophoricoside in histamine-induced scratching behavior. As shown in Figure 2, orally administered sophoricoside inhibited the scratching behaviors by 47.31% (Figure 2B). Terfenadine was used as a positive control in this study.

### 2.2. Effect of Sophoricoside on DNCB-Induced Atopic Dermatitis and IgE Levels in Serum

**Figure 3:**
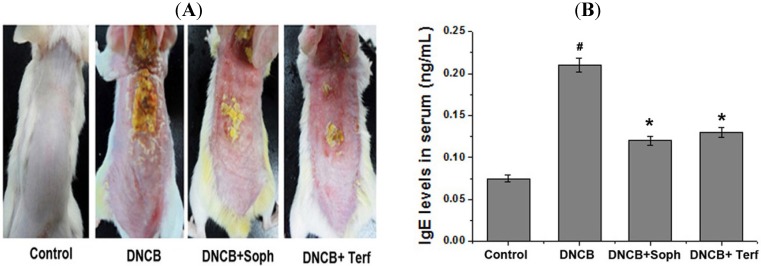
Effect of sophoricoside on the DNCB-induced atopic dermatitis and serum IgE level. (**A**) The BALB/c mice (n = 6) were sensitized with 100 μL of 0.1% DNCB in acetone/olive oil (3:1) or vehicle [acetone/olive oil (3:1)] applied to the dorsal skin twice each week for a total period of 5 weeks. After 3 weeks, sophoricoside (2 mg/kg) was orally administered 2 week prior to the end of the experiment; (**B**) Blood samples were collected and then levels of serum IgE in the indicated groups were measured using ELISA method. Each datum represents the means ± S.E.M. of three independent experiments (^# ^*p* < 0.05 *vs.* control group, * *p* < 0.05 *vs.* DNCB -treated group).

In order to evaluate the regulatory effects of sophoricoside in an atopic dermatitis *in vivo* model, DNCB was administered to BALB/c mice. As shown in Figure 3A, when mice were treated for 2 weeks with sophoricoside, the atopic dermatitis was recovered to a significant extent. To evaluate the effects of sophoricoside on IgE levels in serum, blood samples were collected. The levels of IgE were measured via ELISA. The results showed that IgE levels were increased as the result of DNCB exposure, but this phenomenon was significantly reduced in the sophoricoside group (Figure 3B).

### 2.3. Effect of Sophoricoside on the Histamine Release in PMACI-Stimulated HMC-1 Cells

**Figure 4:**
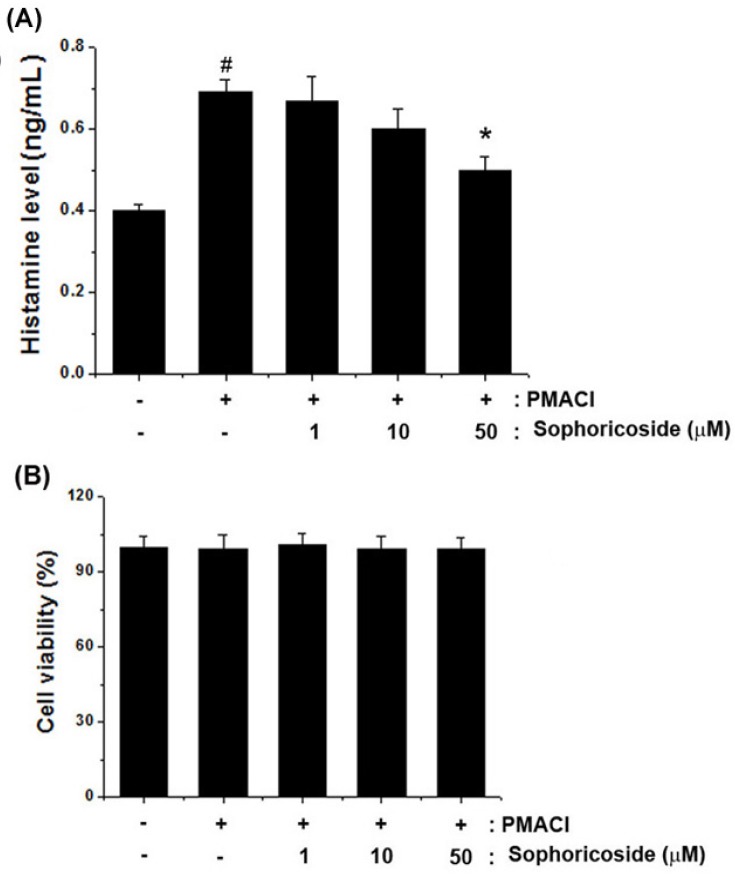
Effect of sophoricoside on histamine release from in PMACI-stimulated HMC-1 cells. Cells (6 × 10^5^ cells/well) were pretreated with various concentrations of sophoricoside (1–50 µM) for 1 h and then treated with PMACI (50 µM PMA + 1 µg/mL A23187) for 4 h. (**A**) The histamine content was measured by the histamine assay kit as described under material and methods; (**B**) Cells (3 × 10^5^ cells/well) were pretreated with various concentrations of sophoricoside (1–50 µM) for 1 h and then treated with PMACI for 12 h. Cell viability was evaluated by a MTT colorimetric assay. All data were represented in the mean ± S.E.M. of triplicate determinations from triplicate separate experiments (^# ^*p* < 0.05 *vs.* control, * *p* < 0.05 *vs.* PMACI alone).

To investigate regulatory effects of sophoricoside on histamine release from mast cells, we measured histamine levels via a histamine assay kit. The results showed that sophoricoside (50 µM) significantly inhibited the PMACI-induced histamine release. The inhibition rate reached up to 30.24% (Figure 4A). Additionally, we observed that sophoricoside did not affect cell viability (Figure 4B).

### 2.4. Effect of Sophoricoside on the Inflammatory Cytokines Production in PMACI-Stimulated HMC-1 Cells

**Figure 5:**
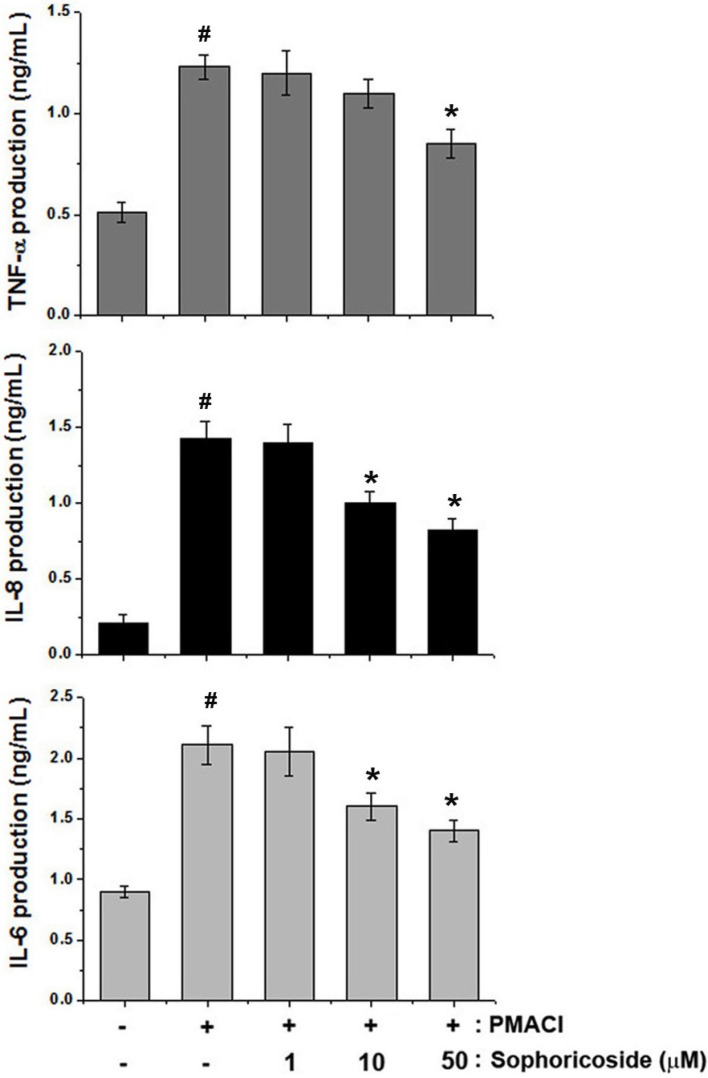
Effects of sophoricoside on the production of inflammatory cytokines in PMACI-stimulated HMC-1 cells. Cells (5 × 10^5^ cells/well) were pre-treated with sophoricoside (1–50 µM) for 1 h and then stimulated PMACI (50 µM PMA + 1 µg/mL A23187) for 12 h. The levels of inflammatory cytokines (TNF-α, IL-8 and IL-6) were measured from cell supernatant using ELISA. All data were represented in the mean ± S.E.M. of triplicate determinations from triplicate separate experiments (^# ^*p* < 0.05 *vs.* control, * *p* < 0.05 *vs.* PMACI alone).

In an effort to determine the molecular mechanism of sophoricoside, the human mast cell line, HMC-1, was employed in this study. We determined whether sophoricoside modulates the PMACI-induced production of TNF-α, IL-8 and IL-6. The levels of TNF-α, IL-8 and IL-6 in culture supernatants were measured via ELISA. As is shown in Figure 5, the production of TNF-α, IL-8 and IL-6 in response to PMACI was inhibited as the result of pre-treatment with sophoricoside in a dose-dependent manner. The maximal rates of TNF-α, IL-8 and IL-6 inhibition by sophoricoside (50 µM) were approximately 31.42%, 43.43% and 34.24%, respectively. 

### 2.5. Effect of Sophoricoside on NF-κB Activation in the Nuclei of PMACI-Stimulated HMC-1 Cells

**Figure 6:**
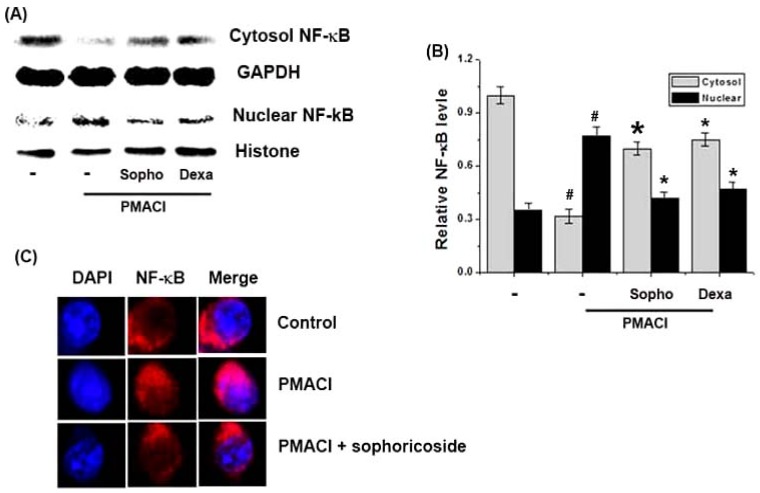
Effect of sophoricoside on the NF-κB activation in the nuclei of PMACI-stimulated HMC-1 cells. Cells(6 × 10^6^ cells/well) were pre-treated with sophoricoside (50 µM) for 1 h and then stimulated with PMACI (50 µM PMA + 1 µg/mL A23187) for 2 h. Dexamethason (50 µM) was used as a positive control. (**A**) Nuclear and cytosol extracts were prepared as described in the *Materials and Methods* section and evaluated for RelA/p65 via Western blot analysis; (**B**) The relative levels of NF-κB were represented; (**C**) The cells were fixed, and stained with TRITC-conjugated phalloidin (NF-κB, red) and DAPI (nuclear, blue), and examined under an Olympus microscope (Magnification ×100). All data were represented in the mean ± S.E.M. of triplicate determinations from triplicate separate experiments (^# ^*p* < 0.05 *vs.* control, * *p* < 0.05 *vs.* PMACI alone).

As the suppression of NF-κB activation has been linked with anti-inflammatory activity, we theorized that the effects of sophoricoside might be mediated, at least in part, via the suppression of NF-κB activation. Additionally, because NF-κB activation requires the nuclear translocation of the RelA/p65 subunit of NF-κB, we evaluated the effects of sophoricoside on the nuclear and cytosol pool of RelA/p65 protein via western blot analysis. In PMACI-stimulated cells, the levels of Rel/p65 in nuclear were increased, but sophoricoside reduced these enhanced nuclear levels of Rel/p65 (Figure 6A). The relative levels of NF-κB (in nucleus) were represented in Figure 6B. The rates of the levels of Rel/p65 inhibition in nuclear by sophoricoside (50 µM) was approximately 50.14%. Figure 6C displays the results of immunofluorescence staining for the distribution of NF-κB (red). PMACI induced translocation of NF-κB into the nucleus, but sophoricoside inhibited this phenomenon.

### 2.6. Effect of Sophoricoside on Caspase-1 Activation in PMACI-Stimulated HMC-1 Cells

**Figure 7:**
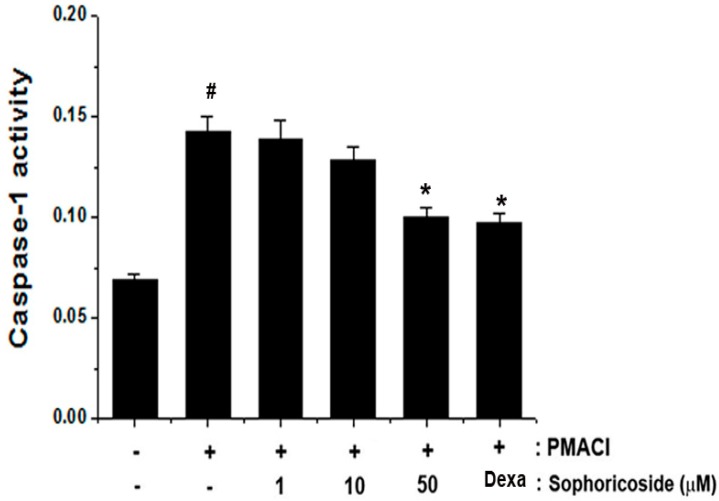
Effect of sophoricoside on caspase-1 activation in PMACI-stimulated HMC-1. The cells were pretreated with sophoricoside (1–50 µM) for 1 h prior to PMACI stimulation for 4 h. Dexamethasone (50 µM) was used as a positive control. The enzymatic activity of caspase-1 was tested by a caspase-1 colorimetric assay. All data were represented in the mean ± S.E.M. of triplicate determinations from triplicate separate experiments (^# ^*p* < 0.05 *vs.* control, * *p* < 0.05 *vs.* PMACI alone).

Activation of caspase-1 induces inflammatory response via stimulation of inflammatory cytokines [15]. In order to determine the regulatory mechanism of sophoricoside on allergic inflammation, we evaluated the effects of sophoricoside on PMACI-induced caspase-1 activation. We showed that the enhanced caspase-1 activity induced by PMACI was significantly reduced by sophoricoside in a dose-dependent manner (Figure 7). 

### 2.7. Discussion

AD is a chronic inflammatory skin disease and is characterized by erythema, edema, and scaling [16]. Generally, steroid therapy is a crucial factor in the treatment of AD, but it cannot be administered over the long-term, owing to its deleterious side-effects. Therefore, several researchers have attempted to find a new drug, which is effective in the treatment of AD [17]. AD was characterized by a potent skin inflammation associated with an elevated level of IgE against many types of allergens [18,19]. On the basis of these studies, we have focused to evaluate the effects of sophoricoside on DNCB-induced allergic reactions *in vivo.*

The effect of sophoricoside on DNCB-induced dermatitis was reported recently. Lee *et al.* [20] showed the effect of sophoricoside on NF-kB signalling in B cells and ear swelling in mice. Our study focused to evaluate the effects of sophoricoside on mast cell-mediated allergic inflammatory response. The findings of this study revealed that sophoricoside significantly reduced DNCB-induced atopic dermatitis. Additionally, sophoricoside caused a reduction in IgE levels in serum induced by DNCB. These results demonstrate sophoricoside’s potential effect on anti-allergic responses via the regulation of IgE levels. 

In pathological skin conditions, histamine is involved in the induction of itching and edema [21]. This study focused on the manner in which sophoricoside regulate the levels of histamine from mast cells and histamine-induced scratching behaviors in mice. We showed that sophoricoside inhibited the histamine release and histamine or compound 48/80-induced scratching behaviors in mice. 

To gain further insights into the mechanisms of sophoricoside -mediated inhibition of PMACI-induced inflammatory mediators (TNF-a, IL-6 and IL-8), we examined the regulatory effect of sophoricoside on intracellular signaling molecules involved in the PMACI signaling pathways in HMC-1. Mast cells play differential roles in the inflammation by initiating and regulating immune responses by the release of various cytokines and chemokines via differential intracellular signal transduction pathways [22]. In response to different stimulation, mast cells release an array of cytokines especially TNF-α, IL-6 and IL-8 with the potential to cause inflammation [23]. Therefore, the development of new biological therapies for inflammatory diseases has generally focused on the blockage of members of the inflammatory cascade, such as cytokines. Cyclosporin A has been employed previously in the treatment of atopic dermatitis, owing to the suppression of IL-6 and IL-8 production noted in cases of severe pediatric atopic dermatitis [24]. In this research, we demonstrated that sophoricoside inhibited the secretion of TNF-a, IL-6, and IL-8 in PMACI-simulated HMC-1 cells. These results demonstrate that sophoricoside exerts an anti-inflammatory effect via the regulation of inflammatory cytokine production. 

The production of these cytokines is associated with increased activation of the gene transcription regulators NF-κB [25]. After a variety of stimuli, the IκB proteins are phosphorylated, and degraded, allowing for NF-κB to translocate into the nucleus where it can bind specific DNA sequences located in the promoter regions of target genes and activate gene transcription, thereby indicating its pivotal function in the regulation of inflammatory responses, via the control of the transcription of inflammatory cytokine genes. From this, inhibition of NF-κB activation has been suggested as an anti-inflammatory strategy in AD. Therefore, we attempted to determine whether the anti-inflammatory effect of sophoricoside is through the regulation of NF-κB activation. The results demonstrated that sophoricoside inhibited the NF-κB translocation into nucleus in stimulated HMC-1 cells. Additionally, we confirmed the effect of sophoricoside on NF-κB translocation into nucleus via immunofluorescence staining for the distribution of NF-κB. Therefore, we hypothesized that sophoricoside might exert anti-inflammatory effects via NF-κB activation. Although sophoricoside attenuated the activation of NF-κB, the effect of sophoricoside on the pathways involving NF-κB (phosphorylation of IκB-α and IKK activation) was not determined. Therefore, further studies will be necessary in order to clarify more precisely the role of sophoricoside on the NF-κB pathway in mast-cell mediated inflammation. 

**Figure 8:**
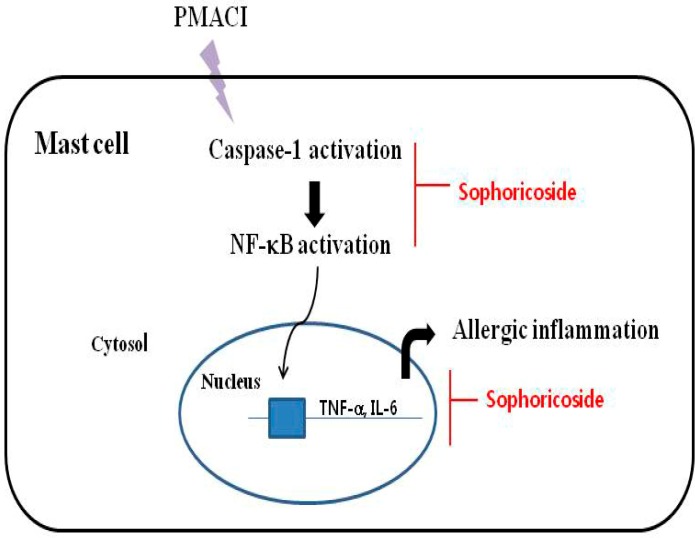
Proposed anti-inflammatory mechanism of sophoricoside in HMC-1.

Caspase-1 is involved in inflammation. The activation of caspase-1 causes IKK phosphorylation and IκB-α degradation. Thus, released NF-κB translocates to the nucleus, where it induces gene transcription [26]. Therefore, we postulated that sophoricoside mediates its effects at least partly through suppression of caspase-1 activation. In this research, we noted that sophoricoside suppressed the PMACI-induced activation of caspase-1. This finding demonstrated that the inhibitory effects of sophoricoside on mast cell-mediated inflammation might derive from the regulation of caspase-1 activation. These results suggested that down-regulation of caspase-1 by sophoricoside might suppress NF-κB activation, and then ultimately suppressed the levels of inflammatory cytokines (Figure 8).

## 3. Experimental

### 3.1. Reagents

Compound 48/80, PMA, calcium ionophore A23187, avidin peroxidase (AP), 3-(4,5-dimethylthiazol-2-yl)-2,5-diphenyltetrazolium bromide (MTT), and DNCB were purchased from Sigma Chemical Co. (St. Louis, MO, USA). Iscove's Modified Dulbecco's Media (IMDM) was purchased from Gibco BRL (Grand Island, NY, USA). Fetal bovine serum (FBS) was purchased from JR Scientific, Inc. (Woodland, CA, USA). Anti-human TNF-α/IL-6/IL-8, recombinant TNF-α/IL-6/IL-8, biotinylated TNF-α/IL-6/IL-8, anti-mouse IgE, recombinant IgE and biotinylated IgE were purchased from Pharmingen (San Diego, CA, USA). NF-κB, and histone antibodies (Abs) were purchased from Santa Cruz Biotechnology, Inc. (Santa Cruz, CA, USA). 

### 3.2. Isolation and Identification of Sophoricoside

The dried *Sophora japonica* was purchased from the Omniherb Company (Daegu, Korea) and voucher specimens (HMP-1201) were deposited at the herbarium of the Daegu Haany University. The fruits of *Sophora japonica* (1.2 kg) were extracted with 80% aqueous methanol (MeOH, 2 L × 3) at room temperature for 24 h and subsequently filtered a filter paper. The filtrate was concentrated in vacuo. The MeOH extracts was dissolved in water (2 L) and then successively partitioned with ethyl acetate (EtOAc, 2 L × 3) and *n*-butanol (*n*-BuOH, 2 L × 3). They were concentrated to afford the residues of EtOAc (9.42 g, SFE), *n*-BuOH (81.9 g, SFB) and the water fractions. The *n*-BuOH fraction (81 g) was applied to a SiO_2_ chromatography column (Ø 10 cm × 20 cm) and eluted with CHCl_3_–MeOH (5:1⟶4:1⟶3:1). The eluted solutions were monitored by TLC and produced 11 fractions (SFB-1 to SFB-11). Fraction SFB-7 (24 g) was purified using an silica gel column chromatography (SiO_2_ c.c) (Ø 10 cm × 15 cm) and eluted with CHCl_3_–H_2_O (65:3510) to produce sophoricoside. The ^1^H-NMR (400 MHz) and ^13^C-NMR (100 MHz) spectra were recorded using a Varian Unity Inova AS-400 FT-NMR spectrometer (Palo Alto, CA, USA).

### 3.3. Animals

The original stock of male ICR mice (5 weeks, 25–30 g) and BALB/c mice (5 weeks, 19–20 g) were purchased from Orient Co., Ltd, a branch of Charles River Laboratories (Seoul, Korea). Animals were housed 10 per cage, allowed access to water and food ad libitum, and maintained at a constant temperature (24 ± 1 °C) and humidity (60 ± 10%) under a 12-h light/dark cycle (light on 08:00–20:00 h). Animal experimental procedures were approved by the ethics committee of Daegu Haany.

### 3.4. Scratching Behavioral Experiment

Before the experiment, the ICR mice (n = 6) were put into acrylic cages (22 × 22 × 24 cm) for about 30 min for acclimation. The behavioral experiments were performed according to the method of Sugimoto *et al.* [27]. The rostral part of the skin on the back of mice was clipped, and compound 48/80 (50 µg/kg) or histamine (100 µg/kg) for each mouse was intradermally injected. The scratching agents were dissolved in tween 80 and then used. Control mice received a Tween 80 injection in the place of the scratching agent. Immediately after the intradermal injection, the mice (one animal/cage) were put back into the same cage; and for the observation of scratching. Scratching of the injected site by the hind paws was counted and compared with that of other sites, such as the ears. Each mouse was used for only one experiment. The mice generally showed several scratches for 1 s, and a series of these behaviors was counted as one incident of scratching for 30 min. Sophoricoside (2 mg/kg) was orally administered 1 h before the scratching agents.

### 3.5. DNCB-Induced Atopic Dermatitis

Experiments were conducted in accordance with a previously described protocol [28].The dorsal skin of the BALB/c mice (n = 6) was shaved and treated with a depilatory prior to the experiment. The mice were sensitized with 100 μL of 0.15% DNCB in acetone–olive oil (3:1) applied to the dorsal skin twice per week for 5 weeks. Control mice received vehicle (acetone/olive oil = 3:1). After 3 weeks, sophoricoside (2 mg/kg) was orally administered 2 weeks until the end of the experiment. 

### 3.6. Cell Culture

Human mast cell line, HMC-1, cells were grown in IMDM medium supplemented with 100 IU/mL penicillin, 100 µg/mL streptomycin, and 10% heat-inactivated FBS at 37 °C in 5% CO_2_.

### 3.7. MTT Assay

To test the cell viability by each concentration of sophoricoside, the MTT colorimetric assay was performed. Briefly, HMC-1 cells (3 × 10^5^ cells/well) were incubated with sophoricoside (1–50 µM) for 12 h. After the addition of MTT solution, the cells were incubated at 37 °C for 4 h. The crystallized MTT (formazan) was dissolved in dimethyl sulfoxide and measured the absorbance at 540 nm.

### 3.8. Histamine Assay

Cells were incubated with the sophoricoside for 1 h, and then incubated with PMACI for 2 h. The reaction was stopped by cooling the tubes in ice. The cells were separated from the released histamine by centrifugation at 400 *×g* for 5 min at 4 °C. The histamine levels were measured by ELISA using a histamine assay kit (Neogen Coporation, Lansing, MI, USA) according to the manufacturer’s directions. Duplicate aliquots of supernatant were measured for each sample.

### 3.9. Assay of Cytokines

TNF-α, IL-6, and IL-8 secretion were measured by modification of an enzyme-linked immunosorbent assay (ELISA). 96 well plates were coated with 100 µL aliquots of anti-human TNF-α, IL-6, and IL-8 monoclonal Abs at 1.0 µg/mL in PBS at pH 7.4 and were incubated overnight at 4 °C. After additional washes, 100 µL of cell medium or TNF-α, IL-6, and IL-8 standards were added and incubated at 37 °C for 2 h. After 2 h incubation at 37 °C, the wells were washed and then 0.2 µg/mL of biotinylated anti-human TNF-α, IL-6, and IL-8 was added and again incubated at 37 °C for 2 h. After washing the wells, AP was added and plates were incubated for 30 min at 37 °C. Wells were again washed and ABTS substrate was added. Color development was measured at 405 nm using an automated microplate ELISA reader. A standard curve was run on each assay plate using recombinant TNF-α, IL-6, and IL-8 in serial dilutions.

### 3.10. Preparation of Cytoplasmic and Nuclear Extract

Nuclear and cytoplasmic extracts were prepared as described previously [29]. Briefly, after the cells were activated with PMACI and then washed with ice-cold phosphate-buffered saline (PBS). These cells were resuspended in 60 µL of buffer A (10 mM Hepes/KOH, 2 mM MgCl_2_, 0.1 mM EDTA, 10 mM KCl, 1 mM DTT, and 0.5 mM PMSF, pH 7.9). The cells were allowed to swell on ice for 15 min, lysed gently with 2.5 µL of 10% Nonide P (NP)-40, and centrifuged at 2,000 *g* for 10 min at 4 °C. The supernatant was collected and used as the cytoplasmic extracts. The nuclei pellet was resuspended in 40 µL of buffer B (50 mM HEPES/KOH, 50 mM KCl, 300 mM NaCl, 0.1 mM EDTA, 10% glycerol, 1 mM DTT, and 0.5 mM PMSF, pH 7.9), left on ice for 20 min, inverted and the nuclear debris was spun down at 15000 g for 15 min to remove nuclear debris. The supernatant (nuclear extract) was collected, frozen in liquid nitrogen and stored at −70 °C until ready for analysis.

### 3.11. Western Blot Analysis

Nuclear and cytoplasmic extract combined with an equal volume of sodium dodecyl sulfate sample loading buffer, boiled for 5 min for denaturation. Samples of protein were electrophoresed using 10% sodium dodecyl sulfate–polyacrylamide gel electrophoresis and then transferred to nitrocellulose membrane. The membrane was blocked in 5% skim milk for 1 h, washed and incubated overnight at 4 °C with primary antibodies in 3% bovine serum albumin in PBS. After washing the membranes, the membranes were incubated for 1 h with horseradish peroxidase-linked anti-rabbit immunoglobulin (secondary antibodies). After three washes in PBST/0.1% Tween 20 for 30 min, the protein bands were visualized by an enhanced chemiluminesence detection system according to the recommended producere (Amersham Corp., Newark, NJ, USA). The quantity of protein was evaluated by using a bicinchoninic acid (BCA) protein assay (Sigma).

### 3.12. NF-κB Immunofluorescence

Cells were fixed with 4% paraformaldehyde and incubated with 5% BSA in PBS for 60 min. The preparation was incubated for 1 h at room temperature with NF-κB Abs diluted in 0.1% BSA (1:200). Next, the preparation was washed 3 times with PBS and exposed to the secondary Abs (fluorescein isothiocyanate-conjugated anti-rabbit IgG at 1:200 and 0.1% BSA/PBS) for 60 min. The fluorescent image was viewed using an Olympus confocal microscope (New Hyde Park, NY, USA). 

### 3.13. Assay of Caspase-1 Activity

The enzymatic activity of caspase-1 was assayed using a caspase colorimetric assay kit (R&D System Inc., Minneapolis, MN, USA) according to the manufacturer's protocol. The lysed cells were centrifuged at 14,000 rpm for 5 min. The protein supernatant was incubated with 50 μL reaction buffer and 5 μL caspase substrate at 37 °C for 2 h. The absorbance was measured was measured using a plate reader at a wavelength of 405 nm. Equal amounts of the total protein from each lysate were quantified using a BCA protein assay*.*

### 3.14. Statistical Analysis

The experiments were shown a summary of the data from at least-three experiments and presented as the mean ± S.E.M. Statistical evaluation of the results was performed by independent *t*-test. A value of *p <* 0.05 was considered statistically significant.

## 4. Conclusions

Sophoricoside can regulate the allergy response *in vivo,* including in compound 48/80 or histamine-induced scratching behaviors and DNCB-induced atopic dermatitis in mice. Additionally, we demonstrated in this study that the anti-inflammatory activities of sophoricoside could be attributed, at least in part, to the inhibition of histamine and inflammatory cytokine production (TNF-α IL-8 and IL-6). These effects of sophoricoside are attributed to the inhibition of PMACI-induced the activation of NF-κB and caspase-1 activation in mast cells. These results provide experimental evidence demonstrating that sophoricoside may prove useful in the treatment of allergic inflammatory diseases.
